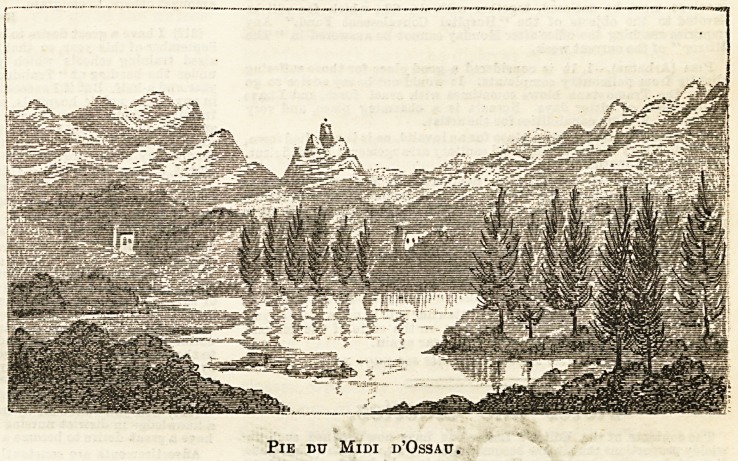# "The Hospital" Nursing Mirror

**Published:** 1899-02-18

**Authors:** 


### "The Hospital" Nursing Mirror.

Being the Nursing Section of "The Hospital."

[Contributions for this Section of "The Hospital" should be addressed to the Editor, The Hospital, 28 & 29, Southampton Street, Strand, London, W.C., and should have the word "Nursing" plainly written in left-hand top corner of the envelope.]

### News from the Nursing World.

### THE QUEEN'S GIFT.

The Queen has just given a further proof of the interest she takes in the health of her subjects at home and abroad. By her command Sir Fleetwood Edwards has forwarded to the Hon. Mrs. Neville Lyttelton, hon. treasurer of the Up-Country Nursing Association for Europeans in India, a donation of £30 to the funds of the association. This is in response to the special appeal recently issued to supply eight more nurses for the work of the association in different parts of India. Our readers may remember that the Queen was reported to have especially commended to Lady Curzon Lady Dufferin's Fund, which is for the benefit of native women and children, so that within a few weeks she has expressed in two very practical ways her affectionate solicitude for the welfare of her people in India both European and native.

### THE NURSES' CONVERSAZIONE.

The Royal British Nurses' Association held its annual reunion at the Royal Institute of Painters in Water Colours, Piccadilly, on Monday evening. No happier meeting-place could be found than these fine lofty rooms, with their soft brilliant light and elegant appointments. The collection of pictures now on view appeared to great advantage, and added greatly to the pleasure of all. Many other entertainments were provided. In one room about a dozen at a time seated themselves at a table, and by fitting the grotesque cups of the phonograph on their ears became the unseen auditors at various places of amusement in the metropolis. It was good fun for the spectators to watch the varying expressions on the listening faces, which passed from grave to gay, and back again across an underlying one of solemn intentness, for which there seemed no cause. In the reception-room Pitman's blue Viennese Band gave a selection of good music, which contributed much to the general enjoyment, although only one or two seemed to listen quietly to it, most of those present being much occupied in renewing old acquaintances and making new ones, or investigating the marvels of the "mutoscope." In the large room beyond an entertainment was provided, which included songs by a "Pierrot Group," a display of animated pictures by Mr. David Devant, and an exhibition of his famous shadowgraphs. Amongst the pictures one, representing the irresistible waves of a rising tide rushing into a sea cave, was really beautiful, and won a well-merited burst of applause. The scene in the rooms was bright and varied. The nurses' costume never shows to greater advantage than grouped together in the distinctive colours of the different institutions, and relieved by other dresses. About 420 were present, a number considerably under that of last year.

### RECEPTION AT VICTORIA PARK HOSPITAL.

On Tuesday afternoon Sir Edward Sassoon, the treasurer of the Hospital for Diseases of the Chest, Victoria Park, and Lady Sassoon gave a reception at the hospital. The corridors on the ground floor and the new nurses' sitting room were made gay and comfortable with a carpeting of red drugget, whilst small tables, spread with tempting cakes and biscuits, provided the usual afternoon refreshment. Music was given by performers who were conspicuous by their white satin dresses. It was by no means a bad idea to arrange an entertainment in the unoccupied ward of the hospital. There is something pathetic in silent corridors and white muffled beds, especially when there are so many patients ready for them when money is forthcoming. The chairman of the committee, in a short speech, said how much the position of the hospital had improved during the year, and how glad they were when Sir Edward Sassoon last year accepted the position of treasurer. They were even more fortunate this year because of Lady Sassoon's presence. Sir Edward Sassoon, in reply, remarked that prosperity was relative, and that the financial position of the hospital, in spite of its great progress during the past year, was grave. It was a blot on the wealthiest city in the world that such a perfect hospital for the purpose, and one so much needed, should languish for want of funds. Lady Rothschild was present for a short time, and will hold a meeting of the newly-formed Ladies' Committee at her own house shortly.

### ST. PATRICK'S NURSES' HOME.

St. Patrick's Nurses' Home is one of the oldest, if not the oldest, of district nursing societies. Most interesting is the account of its foundation and its subsequent struggles for existence. Nearly twenty-three years ago four ladies, whose work amongst the poor led them to employ a district nurse for the sick, banded themselves together to support her. Nurse after nurse was added to the staff, every means that could be devised was used to raise the necessary money, until to-day a staff of 14 nurses are housed in a comfortable home, and it is a recognised training school for the Queen Victoria's Jubilee Institute. It has a very respectable alance in hand, after an expenditure of £1,493. The annual meeting, which was held the other day in the Molesworth Hall, Dublin, was attended by Lady Cadogan and many eminent citizens and sympathisers. His Grace the Archbishop of Dublin presided, and in the course of his remarks said that the nurses' work was solving one of the most pressing social problems of the day. Amongst those who spoke on behalf of the institution were Mr. J. H. Campbell, Q C., M.P., and the Presidents of the Royal Colleges of Surgeons and Physicians. St. Patrick's Nurses are divided into two branches, the one the permanent staff, and the other the probationers—already trained nurses—undergoing their course of instruction in district work. St. Patrick's Home is an institution conducted in accordance with the principles of the Church of Ireland, though no restriction of creed is imposed upon patients. The Catholic Home was established some years later, and is called the St. Lawrence.

### RECOGNITION OF SERVICE.

THE Liverpool Hospital Saturday Fund has been considerably augmented of late years by the "ladies collection," of which the two honorary secretaries have been Miss Lewis, matron of the Stanley Hospital, and Miss Darlington. Both ladies (obliged by circumstances beyond their control) have resigned, and their places have been filled by Mrs. Pagan and Miss Cunningham. The ladies of the committee, in order to express the recognition in which the services of the outgoing secretaries were generally held, organised a social gathering, at which an enjoyable musical programme was provided; and, in the course of the evening, Mrs. Kellett Smith and Mrs. Glover presented Miss Lewis with a gold chain-bracelet, and Miss Darlington with a writing cabinet. The gifts were a surprise to their recipients, who were much touched by them. These ladies still retain their places on the Executive Committee, and it is hoped that by perfecting the organisation to which they belong, £1,000 a year will be added to the resources of the charity.

### THE NURSES' CO-OPERATION.

THE year just closed has been a most prosperous one to the Nurses' Co-operation, 8, New Cavendish Street, W. The number of nurses on the staff stands at 466, and their gross earnings at £38,149—something more than £75 a piece on an average. After deducting expenses of management from the 7½ per cent. commission accruing to the society for that purpose, £1,383 has been paid into the capital account. Two nurses have been distinguished during the year. Nurse Geddes received the Order of the Royal Red Cross from Her Majesty, in recognition of her services in Egypt; and nurse J. E. Wheatley has been admitted to the ranks of the Honorary Serving Sisters of the Order of St. John of Jerusalem in England for plague work at Poona. The new residential home for which the Dowager Lady Howard de Walden has so generously given the site and a considerable portion of the building funds, is now in the contractor's hands, and the completion is promised in the course of the next 18 months. The plans have been carefully drawn by her ladyship's own architect, and she herself has shown practical interest in every detail. Miss Hicks, the first superintendent and organiser of the co-operation, has been instrumental in obtaining the desirable development. Miss Amy Hughes, her successor as superintendent, has succeeded admirably in the discharge of the duties, and in winning the confidence of the nurses. The annual ordinary general meeting of the members was held on February 7th.

### HEREFORD NURSING ASSOCIATION.

THE annual meeting of the subscribers of the Hereford Nursing Association was held recently. The Mayor, who presided, said that the financial position of the association was sound, but that there was no reason why the balance in hand should not be larger. A balance of £150 was not too much, as in the event of an epidemic it would be required. The report stated that there had been no change in the nursing staff, and that one hundred fewer cases had been attended than during the previous year.

### A MUNIFICENT BEQUEST.

THE sum of £3,000 is a very satisfactory nest-egg for a nursing fund. The inhabitants of Maidstone, who require the constant services of a nurse, and who are too poor to pay for one, will for the future have one provided from the bequest of the late Mr. Samuel Bentley. The committee of the West Kent Hospital will have the administration of this fund, which will be kept distinct from the hospital accounts. The nurses will be supplied when needed through the Stephen Monckton Nurses' Home. The fund will doubtless be a useful one, and supplement the work of the district nurses, who do not supply constant attendance.

### THE NURSING INSTITUTE, SUNDERLAND.

LAST year has been the most successful in the annals of the Nursing Institute, Sunderland. The Christmas festivities were made the occasion of presenting each nurse with a bonus—the first the committee has been able to afford. Some of the nurses received £6, and none got less than £2. The matron is in good spirits about the future of the Institute, and hopes for still better things next year. She is proud of her nice nurses, and takes a personal interest in each. She is also anxious to get thoroughly good sensible nurses upon her staff, who are not afraid to turn their hand to little useful things when the need arises.

### WELSHPOOL VICTORIA NURSING INSTITUTE.

THE first annual meeting of the Welshpool Victoria Nursing Institute has recently been held at Welshpool. Mr. W. Forrester Addie, chairman of the committee, presided, and the vice-president, Miss F. E. Howell, who takes much interest in the society, was present. The institute was established to commemorate Her Majesty's Diamond Jubilee, and is affiliated with Queen Victoria's Jubilee Institute for Nurses. It is warmly supported by the medical men of the town, and Nurse Crabbe has attended 84 cases, to most of whom she had been sent by the doctors. The finances also flourish; the capital of the permanent fund stands at £110, and that of the general fund is £27, whilst Earl Powys has granted the house in which the institute and emergency wards have been established on very favourable terms.

### SHORT ITEMS.

A BRILLIANT ball was given at Darlington in aid of the Hospital and Queen's Nursing Association. Lord Barnard and the Lady Mayoress received the guests, who numbered 300.—The Barry District Council has made a grant of £400, to be paid in twelve monthly instalments, to the Barry Nursing Association and Accident Hospital, on the condition that five beds are provided.—At the annual meeting of the Dublin branch of the British Medical Association, the President of the College of Physicians (Dr. J. W. Moore) moved: "That all sanitary authorities should issue leaflets pointing out the dangers of infection (in tuberculosis), and giving directions for avoiding those dangers, and the branch call upon Queen Victoria's Jubilee District Nurses to aid in distributing such leaflets."—The large number of 350 cases had to be refused last year by the Leeds Trained Nurses' Institution because of there being no nurses free to undertake them, yet the staff numbers 99.—The sum of £350 has been paid to the trust fund of the Institute of the Royal National Pension Fund for Nurses.—The West Kent Hospital authorities have spent £1,120 in the purchase of a house adjoining the hospital for the accommodation of the nurses attached to it.

### Hints on the Home Nursing of Sick Children.

By J. D. E. Mortimer, M.B., F.R.C.S., formerly Surgical Registrar, &c., at the Hospital for Sick Children, Great Ormond Street.

(*Continued from page* 201.)

### SPECIFIC FEVERS—SCARLET FEVER AND DIPHTHERIA—ENTERIC (TYPHOID) FEVER.

### Specific Fevers.

For reasons already mentioned I must refer the reader to works on nursing in general for information about the usual course and management of these cases. The subject is also treated in detail by Dr. Hardman ("Fever Nursing"). There are, however, a few points in regard to young children requiring notice. I would warn the nurse that as they are apt to be suddenly attacked while in their usual health, she must be prepared for much restlessness and quite excusable fretfulness. Diet should be mainly milk diluted with barley water, &c. Feverish children do not as a rule care for beef-tea, and meat essences, jellies, &c., are not only unnecessary in most cases but harmful, as they throw additional work on the liver and kidneys just at a time when these should not be disturbed. Plain cold water in moderate quantity should not be forgotten, with apple water, lemonade, &c. If the child is not old enough to be trusted with a piece of ice to suck, this may be twisted in a corner of a handkerchief held by the nurse (the pain and irritation of teething may often be so allayed). It is always important, especially in diphtheria and enteric fever, to cleanse the mouth and teeth with a soft brush or rags dipped in boracic or similar lotion, or by syringing; the spaces between the cheeks and gums must be carefully attended to.

No dogs, cats, or other pets should be allowed in the sick rooms, and any toys, books, &c., should be either destroyed or sent to a fever hospital (with an explanatory note or label on the inner covering of the parcel). They can seldom be satisfactorily disinfected. The nurse should see that all clothing, &c., which the child had in use at the time of the onset, or shortly beforehand, is disinfected or otherwise dealt with. It not infrequently happens that things are put away in a drawer and forgotten till the child has recovered, and being then brought out the infection is given to some other member of the family. Many cases which occur after patients return home from fever hospitals can be so accounted for.

### In Cases of Scarlet Fever and Diphtheria there may be much resistance to applications to the throat. These are sometimes not so necessary as to be worth fighting over, or although desirable, persistence may do more harm than good by the child becoming resentful or exhausted. Just as when difficulties arise in giving medicine to a child, the doctor should be asked to decide whether the treatment is to be carried out thoroughly or left alone altogether. It may be possible for a nurse to do this work singlehanded by wrapping the child firmly in a blanket or sheet with the arms inside, and holding the head between her knees, but a struggle between a nurse and patient is obviously most undesirable, and she should have proper assistance whenever it can be obtained. If force is necessary for medicine-giving and feeding as well, let all be done together as far as possible.

In scarlet fever I prefer eucalyptus oil to carbolized for inunction; the latter may, especially in young children, produce bad effects by absorption, and its antiseptic power is not such as to make up for the risk.

### Diphtheria.

Probably in no disease is there more need for unremitting diligence and intelligent observation, and the younger the child the more this is to be borne in mind. Any change in the character of the voice or cough, any increased signs of laboured breathing, any alteration in colour, should be notified to the doctor without delay, and later on the nurse must be on the watch for any sign of paralysis, such as choking during swallowing or a nasal twang in the voice. An accurate account must be kept of the times of feeding and the amount taken. The nurse must not be surprised if the tent and steam kettle are not ordered, even in tracheotomy cases, as there are certain disadvantages, such as confinement of air, chill and discomfort from condensation of steam, difficulty in getting at the child, which have led many eminent physicians and surgeons to discontinue using them.

A child on whom a tracheotomy has been performed must never be left for an instant until breathing by the natural passage is completely re-established.

Hence in private houses, more especially when on night duty, the nurse must see that she has efficient means of communication with others in case anything is wanted, and that proper arrangements have been made for summoning the doctor if occasion arises; also that she is well provided with fuel, ice, stimulants, milk, &c., &c. The clothes near the tube should be protected by waterproof material or Gamgee tissue, but the nurse must not rely on the child coughing out the secretion. She must be ready to catch it with a swab or feather as it appears at the opening before it is sucked back. When the time arrives for the tube to be taken out the nurse should, in the doctor's presence, replace it or use a dilator. Her success will not only be satisfactory to others, but will give her confidence to act with promptitude if suddenly required to do so. "Sucking at a tracheotomy tube is as unsurgical as it is dangerous, and has caused the unavailing sacrifice of many an heroic life" (Edmund Owen). Later on, the muscles of respiration may be affected by paralysis with liability to failure of the heart, and if so the child must on no account be allowed to change position suddenly, especially to sit up in bed. During convalescence over-exertion and over-excitement must always be avoided.

### Enteric (Typhoid) Fever in children usually runs a milder course than in adults. The onset may be comparatively sudden; there is often little if any rash, and diarrhœa may not be a prominent symptom or may be entirely absent. As regards the management, the same rules are necessary as in older patients. Attention to cleanliness without unnecessary disturbance, precautions against bedsores, strict regularity and limitation of diet, careful watching, especially at night, and efficient disinfection of the excreta, of any contaminated articles, and of the nurse's hands, are, of course, essential. It may be mentioned that apparent dislike to food may be due to sore throat. Enemata may be needed on account of constipation, and if so must be given with even more than usual care, lest an ulcerated bowel become perforated. In mild cases and when the worst is over it is well to vary the dietary as much as possible by giving Benger's food, thin arrowroot, blancmange, cocoa, malt extract, beaten eggs, jelly, orange juice—in fact anything which contains no particles likely to irritate and does not tax the digestion. This must, of course, be done under medical approval. The doctor should be asked for instructions to meet sudden emergency, such as serious hæmorrhage or signs of perforation of the bowels. In these cases it is important that the child should at any rate lie as still as possible. When recovering, the child must not sit up or get up too suddenly, as this may cause faintness.

(*To be continued.*)

### The Nurse's Part in the Prevention of Tuberculosis.

THE largest and most enthusiastic audience that has ever attended a sessional lecture of the Royal British Nurses' Association assembled at the rooms of the Medical Society, 11, Chandos Street, W., on Friday, February 10th, to hear Dr. Bezly Thorne deliver his address on "The Nurse's Part in the Prevention of Tuberculosis." Dr. Bezly Thorne first remarked that instead of repeating a former lecture, as he had been requested to do, he had thought it desirable to approach the subject from the point of view of those who may be called upon to carry out the directions of medical men in the course of treatment which is novel in this country, but which was more effective than any other in checking the ravages of a disease now claiming in England and Wales 70,000 lives a year. Contrary to the prevalent view up to the present time, tuberculosis is not only preventable, but is one of the most curable of diseases. He remembered, as a medical student, his own surprise when Dr. Hughes Bennett, of Edinburgh, certified that the post-mortem examination of from 20 to 30 per cent. of cases dying from other complaints showed healed cavities in the lungs which had been undoubtedly caused by tubercular phthisis. If to these unsuspected cases was added the number of known cases, it became apparent that about half the population must at one time or another have been affected with tuberculosis of the lung. For more than a generation past the open-air treatment has been practised in several places in Germany, and within the last seven or ten years it has been introduced into France and Switzerland; but in Great Britain it has remained practically unused up to the present day. The essential details of the open air treatment are: Residence in rooms of sunny aspect, in which there is a free admission of pure fresh air day and night; that the patient should be out of doors in all weathers from eight a.m. until night, excepting for meals and toilet purposes; that verandahs or summer-houses should be available wherein shelter may be obtained from rain, hail, &c., and scorching sunshine. Pleasant surroundings, lounges under shady trees on a lawn, agreeable walks and occupation, such as gardening, are all advantageous. The objects for which the patient is kept in fresh air are that he may draw into his air passages and lungs pure and uncontaminated air with every breath during the twenty-four hours, in order to promote the health of the respiratory apparatus; to check morbid processes in the lungs; to restrain the proliferation of the micro-organism on which the processes of the disease depends; to aerate the blood fully; and to cultivate a healthy appetite. None of these objects can be attained by segregating patients in the wards of a hospital, and it is of paramount importance to ensure that no phthisical patient inhales his own or another person's breath, even in a diluted state. It is also of the utmost importance that the food should be abundant, pure, and nourishing; it must be well cooked, and administered at such intervals, and in such quantities, as the medical attendant may deem consistent with the state of the patient's digestion. Dr. Bezly Thorne said that it must be clearly stated that tuberculosis is an infection, and not an inheritance, but it must be admitted that there are certain constitutional conditions either inherited or acquired that predispose the subject to it. Still the most delicate individual will not become subject to it apart from the reception of the specific organism into the system. It may be communicated by inoculation through an abrasion in the skin; by inhalation; and by ingestion with food. There is abundant proof that the hand may be affected by coming into contact with discharges containing the specific germ. The habit of expectorating into the fire is very dangerous, for any sputum unconsumed becomes dust and so liberates the germs. Wardmaids have been known to become infected from this cause. Every patient ought to be provided with a spitting flask such as that designed by Dr. Dettweiler, which can be carried in the pocket. Spittoons containing disinfectant should be provided. Neither linen or paper handkerchiefs should be used for the purpose. Poor persons can make and use small paper cones and destroy them immediately after use, preferably by fire. Dr. Thorne traced the effect of infection by inhalation very closely, and passing on to that by ingestion emphasised the importance of sterilising milk by submitting it to 180 degrees of heat, using clotted cream, and avoiding butter, unless from an ascertainedly pure source. Meat, he said, especially beef, must be cooked until the last tinge of pink is on the vanishing point. We have merely indicated the general scope of Dr. Bezly Thorne's lecture, and are unable from want of space to give his lucid explanation of the theoretical aspects of the disease, its propagation by infection, and the way in which the new treatment effects a cure. Nor can we find room for the clear, practical directions for nursing patients. It is hoped that the address may be published in pamphlet form, and thus place in the nurses' hand a very practical and up-to-date guide to the nursing of tuberculosis.

### District Nursing of Infectious Diseases.

THE home nursing of non-infectious diseases is now established throughout the country by means of complete and successful organisations. Only time is needed for the presence of the trained nurse to be regarded as indispensable in every hamlet. The true economy of bringing the nurse to the sick in certain cases instead of the sick to the hospital is becoming more evident daily, and although there must always remain a percentage of cases that can only be adequately treated in hospitals, yet many ailments can be dealt with nearly, if not quite, as well at home, while some must of necessity be treated there. With the growth, however, of the scientific knowledge of bacteria and the laws that govern their propagation and development, infectious diseases are more strictly isolated, and the energies of sanitarians are much occupied with the best means of securing effective isolation for the patients suffering from them. Thus there have been hospitals established for their reception, costly devices for sterilising clothing and houses, and elaborate preparations for quarantine. These to a certain extent meet the difficulty in populous centres, but it is otherwise in sparsely-inhabited districts, and cases still crop up of patients being left to nurse themselves as best they may, or of a whole village becoming victim to an epidemic through the kindly but ill-instructed attentions of their neighbours. As the result of a correspondence between the Duchess of Sutherland, president of the Sutherland Benefit Nursing Association, and the County Convener of Scotland, and of a conference of sub-committees appointed for the purpose by the Public Health Committee and by the Executive Committee of the Sutherland Benefit Nursing Association, an arrangement has been made by which a fully-trained nurse has been added to the staff of the nursing association. Her duty is to nurse cases of infection in whatsoever part of the country they may occur. When she is not thus engaged she is at the service of the association for general nursing. The association receives an allowance of £70 a year for her maintenance from the Committee of Public Health, of which £50 is paid to the nurse as salary, and £20 is retainedto provide lodging (both when without infectious work and whilst nursing a case). The nurse (who has had six years' experience) entered on her duties on November 1st last, and the value of the experiment will therefore soon be known by experience. The first point to be noticed—one which the Duchess of Sutherland has wisely brought into prominence—is the necessity of employing fully qualified nurses for this work. The majority of the women on the staff of the Sutherland Benefit Nursing Association are cottage nurses of six months' training in maternity nursing. Such women are not fitted for the care of the complicated and more serious infectious cases. Medical assistance may not be available for hours, or even days, at a time, and the nurse in attendance must be prepared to act from experience in emergencies, whilst it is only the trained observer who can accurately report the progress of symptoms to the medical man in charge. The cottage nurse may do well enough in maternity work, but nothing less than the best can be safely employed in acute illness. A second important point must also be observed in any scheme of this character, and that is that the nurse's sleeping accommodation must be provided away from her patient's house. It is not safe for the nurse to take her rest in an infected atmosphere. There is yet a third point, and this will probably soon force itself into notice. The unit of the system should be two nurses. In all serious cases night as well as day nursing is essential, and, although every case may not be serious, sooner or later such a one will occur, and the organisation must be prepared to meet the need. It is hardly practical that nurses for non-infectious disease and nurses for infectious disease should occupy the same home when disengaged. Some time must elapse after the nurse leaves her patient before she is out of quarantine, and before she can mix freely with other nurses, of all people. If the scheme prove a success, and there is no reason why it should not if well planned and carried out, it will ultimately resolve itself into the establishment of an entirely distinct branch of the Nursing Association. The staff will be separately housed, and will consist of highly-trained nurses who have experience in the special difficulties of their task. The existence of such a staff of nurses, available at a moment's notice, will undoubtedly be of the utmost value in combating outbreaks of infectious disease.

### The Book World for Women and Nurses.

WILLIE: A STORY OF A CHILDREN'S HOSPITAL. M. CALDERFORD. (Swan Sonnenschein and Co. 1899. 1s.)

"WILLIE'S" story, as told by one of the nurses in a children's hospital, is a very sad one, and has a ring of reality about it which makes it all the more pathetic. The hero of Miss Calderford's little book is introduced to us on his death-bed in the closing days of a life full of many sufferings. His case is an incurable one, and every care and attention is shown to the poor diseased child. Willie was a child of six, who had been admitted to the hospital some three weeks earlier than when the story opens. "He was a poorly-developed, weakly, pallid, emaciated boy. The doctors spoke of him as being tubercular from head to foot. At night he would remain awake for hours tossing about in his cot." It was whilst on night duty one Christmas time, when all round him in the ward were manifestations of seasonable rejoicings, that the child chanced to be the means of an unseen blessing to the night nurse who writes the account. It is only an incident, but one of moment to the woman, and shows how the sorrows of one suffering mortal may be assuaged through the witness of another's pain; and thus it chanced that the child on the bed in physical pain was the unconscious instrument of help to the woman whose suffering was mental. The story is a pathetic one, as we have said, but has its happy side; and Miss Calderford's views of existence, as shown here, being free from the charge of morbidness, prove healthy reading.

### Some Superstitions of Southern India.

(BY A NURSE CORRESPONDENT.)

THE inhabitants of India, like those of all Asiatic countries are very superstitious. A young woman, who had been ill for about three months when I first saw her, asked me very anxiously whether there was any chance of her recovery. On my replying in the affirmative and appearing surprised she gave the following excuse: Her husband's first wife had died, and if she were to die to, people would think him an unlucky man, and so would not give their daughters to him in marriage. This woman read and wrote Mahratti, which was her native language, very well, and helped to keep accounts for her husband. They belonged to the Dhirzi or tailor caste, and could only intermarry with families of the same caste. Misfortunes of this and many other kinds are put down to the anger of some particular god or goddess, and much money is often spent in travelling to the shrine and in offerings. A kind of bargaining often goes on between these people and their divinities. For instance, a child was ill; the mother went to her particular household deity and made a vow that if the child recovered she would bring a goat as a sacrifice. It meant a great deal to her, for she was very poor. It is often so, but I have never known them to break their vows.

To all native minds the "devil" is a very real person indeed. Comparatively few understand a fit, or lockjaw, or, indeed, any kind of brain mischief, believing, as a rule, that the "devil" has entered into the patient, and branding and various other methods of exorcism are resorted to without stint. Branding, indeed, seems to be the remedy for all ills. A dislocation of the arm is painful; and with the full consent of the injured person, and in good faith, the arm is forthwith branded, so that eventually when the case is brought for treatment it is to the burn the first attention must be given.

A native gentleman was once leaving our house, after a visit, and sneezed on his way to the hall-door; to our no small astonishment he turned round and sat down again, asking to be allowed to stay a few minutes longer, as it was very unlucky to leave a house directly after sneezing. He then told us that if he were to meet a funeral on his way home he should consider that to be an omen of great good luck. As he had sometimes to stay with my father for hours on business matters, we were in the habit of supplying him with various spices, fruits, and nuts—anything, in fact, which did not require to be cut with a knife. For instance, he would not eat melon or pomolo (a large fruit) at our home, the reason being that, as he could not eat the whole, he was unable to eat a slice. The knife might at some time or other have been used to cut meat, he said; we were obliged to confess that such a thing was more than probable. He was a high-caste Brahmin, and such a thing would have broken his caste. He was a very wealthy man, but, being childless, he at last adopted a son. His wife could hardly walk when she had all her jewels on, yet, in spite of all the inconvenience they caused her, she frequently asked for more. This woman was not educated at all, and was very much upset about her husband coming to the house of a Christian. When, eventually, he lost a valuable jewel at a fair, she declared that some god was offended with him, and so had punished him by taking away an article of the jewellery he prized so highly.

### Presentation.

ON January 30th the nursing staff of the Park Hospital, Hither Green, Lewisham, presented Nurse Woodbine with a silver-plated steriliser, on her leaving the above hospital to take up the duties of sister of the Princess Christian Hospital, Sierra Leone.

### A Book and its Story.

### PENELOPE'S EXPERIENCES IN SCOTLAND.

It is rare nowadays to meet with a book that is genuinely amusing, upon whose scenes and characters, touched with the light hand of a refined and discriminating satirist, the genial light of humour plays in ever-varying shades; but the gifted authoress of "Penelope's Experiences"* possesses this rare gift, and in consequence her writings have an unusual charm and originality.

Salemina, Francesca, and Penelope, three fair Americans, agree, not for the first time apparently, to become travelling companions. To start on a journey of discovery through Scotland is one of their plans, and what befel them, and their impressions of the country and its customs, as seen by American eyes, form the subject of the "experiences." An oddly-assorted trio enough, but possessed of one valuable secret in "knowing the worst of each other." Penelope gravely asserts also, by the way, that on no conceivable point are they agreed. Modes of travelling, choice of food, amusement, appeal to them from differing standpoints. "This does not sound promising, but it works perfectly well by the exercise of a little flexibility." A *contretemps* occurs at the outset. Having purchased first-class tickets they discover that although the difference in price is considerable, in point of comfort there is little to observe between the two classes, except perhaps that there is a more profuse allowance of buttons in the first-class cushions, and rather less yielding stuffing in those of the third-class carriages. This being the case, Francesca decides at the last moment to change the tickets, having first taken the precaution to bundle her fellow travellers hastily into an adjoining carriage. After a heated encounter with the booking clerk, who at first declines to make the exchange, she returns in triumph with the explanation, "I told him, they were bought by an inexperienced American lady—that's you, Salemina. He replied, 'That the tickets had been stamped on.' I said, 'So should I be if I returned without exchanging them.' This terrible alternative moved the obdurate official, and the exchange was rapidly effected." They pursue their journey and beguile the tedium of the way by the perusal of heavy literature provided by sympathetic friends, so heavy in bulk as to provoke from the guard the inquiry, "Do you belong to these books, ladies?" Abundant amusement is extracted from their pages, the following passage making them duly thankful for present day modes of travelling instead of those of the last century, when by "repairing to Canongate or Holborn passengers could be received in a coach which performed the whole journey in thirteen days, without stoppage (if God permits)." They arrive at Edinburgh in rain and mist, such weather as that which greeted the ill-fated Queen when "she set foot upon her native plain." "The very heavens did manifestlie speak what comfort was brought to this countrie with her, to wit, sorrow, dolour, darkness, and all unpiety, for in the memory of man was never seen a more dolorous face of the heavens than was at her arrival." Nothing, however, can daunt the good spirits of the travellers, although as they alight at the door of "good," or at least, "pretty good," Mrs. McCollop, their landlady, it is too dark for them to discern the outstretched hand of the driver distinctly, but not too dark for him to see "half-a-crown, and demand three shillings as his fare." The glow of warmth and comfort—of luxury even—which greets them from Mrs. McCollop's open door soon dispels the passing discomforts encountered outside. The unanimous verdict is one of surprised delight; "and," adds Francesca, grown wise by previous experiences of landladies and their "little ways," "The tea may be a present from Mrs. McCollop and the sugar not an extra; the fire may be included in the rent, and the piano taken away to-morrow to enhance the attractions of the dining-room floor," but "for the present these joys, substantial if fleeting, are ours," and they rest content.

Susannah Crum, the Scotch maidservant, is an amusing specimen of her class. As a bureau of information she is a failure, for with characteristic and canny obtuseness she replies to all questions with an invariable "I canna'" or "I couldna' say." From their introduction into Edinburgh society by Lady Bond dates a charming romance which ends, after many contrary "episodes" in the happy union of two persons apparently antagonistic in temperament. "The Scotch butler" strikes Penelope as being even more sanctimonious than functionaries of his class in England. Surely the English butler cannot be outdone in solemnity by his Scotch *confrère*, but this sounds rather like it: "He hands your plate as if it were a contribution box, and in his moments of ease when he stands behind his master I am always expecting him to pronounce the blessing." "It would be impossible to deny the key of the wine cellar to a being so steeped in sanctity, but it has been done in rare and isolated cases." At their first dinner party Penelope has the delight of being taken in by a real earl, of whom she modestly avers that "after he had discovered my point of view, he found me delightful." "It always takes an earl a certain length of time to understand me!" But he on his part hardly conveys the impression to the reader when we are told that he wasted much time in watching his radiant *vis a-vis*, Francesca, nor is peace of mind assured to Penelope by hearing Salemina reply to an entranced "W. S." by her side, in reply to an inaudible question, "Miss Hamilton appears simple, but really she is as deep as the Currie Brig." An apt comparison on Salemina's part, but in no wise concerting to Penelope, who desired to pass as the guileless being she appeared to be.

Francesca's impressions of the young Scotch minister who sat by her were the reverse of those formed by her friends. They found "Ronald Macdonald quite the handsomest man in the room!" Francesca may have had her own reasons for declaring him to be "a most condescending, ill-natured prig," for a brisk interchange of amenities had taken place between them on the respective merits of America and Scotland, and Francesca's susceptibilities were ruffled by her neighbour's assertion that "no royal city in Europe could boast the centuries of such glorious and stirring history as Edinburgh." Francesca replies meekly that "Edinburgh did not appear to be stirring at present . . . until you have visited a city like Chicago you can have no idea of push or enterprise!" The minister's reply hardly threw oil on the troubled waters: "Happily, Edinburgh is singularly free from the taint of the ledger and the counting-house; it is Weimar without a Goethe, and Boston without a twang." But in spite of these small differences Francesca buries the hatchet and becomes Mrs. Ronald Macdonald. From the attentions of grave and infatuated professors the trio seek distraction in a picturesque country retreat, and there, amid these charming surroundings, we will leave them.

The authoress has scattered so much smart writing throughout "Penelope's Experiences," tempered with the genial humour already spoken of, that we cordially recommend her book to the large class of readers to whom it will appeal.

* "Penelope's Experiences in Scotland." By Kate and Wiggin. (London: Gay and Bird. 6s.)

### Everybody's Opinion.

[Correspondence on all subjects is invited, but we cannot in any way be responsible for the opinions expressed by our correspondents. No communication can be entertained if the name and address of the correspondent is not given, as a guarantee of good faith but not necessarily for publication, or unless one side of the paper only is written on.]

### TOE POSTS.

"A CORRESPONDENT" writes: Seeing a question in your paper of January 21st (No. 169) of someone wanting to know the address of a boot maker who would make "toe propped boots," I write to say I think "Unfortunate Nurse" would find Mr. Messenger, Evershott Street, Oakley Square, N.W., would make boots such as she wants. He is very clever, and makes comfortably and neatly at the same time for tender or deformed feet. I have suffered from tender feet, and have tried most of the best bootmakers in London, but since I have gone to Mr. Messenger I have never required to go to anyone else.

### HOME FOR NURSES NO LONGER ABLE TO WORK.

"A PRIVATE NURSE" writes: Policy 278 has a very poor opinion of her colleagues if she imagines that their wish to have a shelter in old age means that they intend to sit down to an idle, gossiping existence. No nurse who has really a vocation could do such a thing. But suppose such a home, situated in a centre of hard-worked district nurses, what a boon it would be to have such nurses at hand to volunteer, as health allowed, on such hard-pressed occasions —that would I think, mean "living for others rather than self." I am sorry the thought, put forward so ably by Miss Davis, is allowed to drop here, as it would, indeed, be a help and encouragement to many of our sisters, who have been helping others all their lives, without a thought of self when too old for work, and have found it utterly impossible to "put by" or join the Pension Fund. Many have been hindered from joining knowing how almost impossible it is to get work in these days after about 45, which means ten years at least of poverty before the pension becomes due, with heavy premiums to pay; unless the nurse is absolutely ill and unfitted for work, and can take sick pay, which might cover the premium but would not keep her also.

### THE ENGLISH HOSPITAL, HUELVA, SPAIN.

"A NURSE" writes: Perhaps my readers may know that the Rio Tinto Company possesses a large copper mine about 50 miles up country. The transit of mineral and material is by a private railway, of which Huelva is the seaport and where there are very large workshops and the largest pier in the world. The number of operatives, with an English staff of 100, is 9,000, and, in addition to the above-mentioned, an average of about 100 men are received from the mercantile fleet which comes into the harbour, who fall sick in port. For those of the staff who may be sick and for the men injured in the works the company have provided two hospitals, one in the mines and one in Huelva. The latter hospital is situated in the highest and most healthy part of the town and is surrounded by a large garden of flowers and fruit; among others, quite a large number of orange trees. It consists of two pavilions and an administrative building, on the upper story of which are single-bedded rooms. The pavilions contain 26 beds and in the upper storey eight bed-rooms. The fittings, beds, instruments, &c., are all on the newest principle, but the sanitary arrangements are different from those to be found in England, for there is not a single drain in connection with the building. The washhouse, water closets, &c., are about 50 yards distant from it, and behind these there is a large tank on wheels to which every drop of dirty water is carried and which is removed twice daily. The work consists of nursing Englishmen invalided with malarial fever, &c., and surgical care of men injured in the works at Huelva or sent from the mines to convalesce. For operations, instruments, towels, &c., are boiled, and healing by first intention is the rule. The staff consists of an English doctor, with two English doctors from the mines to consult with whenever necessary, one Spanish doctor and a qualified assistant, one practicante, who is really a male nurse and dresser, an English matron, Miss L. W. Blackadder, certificated from Liverpool Royal Infirmary, and three Spanish female nurses. There are also a cook, housemaid, two laundry maids, message boys, gardener, and night guard.

### Minor Appointments.

KASR-EL-AINY HOSPITAL, CAIRO.—The following Nursing Sisters have been recently appointed to this hospital: (1) In November Miss Margaret Sameon, who was trained and afterwards staff nurse at St. Thomas's Hospital for three years, where she also undertook sister's duties, was appointed. She has also received district training as a Queen's Nurse. (2) In December Miss Dora Barnett was appointed. She was trained for three years at Chester Infirmary, afterwards sister at the Park Hospital, Hither Green, and night superintendent of Chester Infirmary. (3) Miss Jane Maude Lattey was appointed in February. She was trained for three years at Liverpool Royal Infirmary, and for the last two and a half years has been sister of the male surgical floor at Kent and Canterbury Hospital. Miss Edith Coxon was also appointed then to increase the staff. She was trained for three years at the West Kent Hospital, Maidstone.

WARRINGTON UNION WORKHOUSE INFIRMARY.—On December 16th, 1898, Miss Hannah Walkington Browne was appointed Superintendent Nurse. She was trained at Brownlow Hill Workhouse Infirmary for three years. She was subsequently sister at the Poplar and Stepney Sick Asylum, and superintendent nurse at Barnsley Workhouse Infirmary, and afterwards for a year charge nurse at the Samaritan Hospital for Women, and for thirteen months sister of the children's wards of the Throat Hospital, Golden Square; sister for holiday duty at the Hospital for Sick Children, Great Ormond Street; and matron assistant, West Kent Hospital, Maidstone.

GRAVELLY HILL WORKHOUSE INFIRMARY, NEAR BIRMINGHAM.—On January 3rd Miss Mary Elizabeth Walker was appointed Sister of this institution. She was trained and afterwards held other posts at St. Pancras Infirmary, Dartmouth Park Hill. Miss Elizabeth Howlett, who was trained at St. Saviour's Infirmary, East Dulwich Grove, London, S.E., was appointed Sister on January 13th. She has had three and a half years' experience at the Children's Hospital, Sevenoaks, Kent. Miss Lydia J. Comber was appointed Sister here on the 12th January. She was trained at the Central London Sick Asylum, Cleveland Street, W.

GRAYS ISOLATION HOSPITAL.—Mrs. E. M. E. Carlisle Heys has been appointed Matron of this hospital. She was trained at Mill Road Infirmary, Liverpool, and has subsequently held the posts of charge nurse at Gore Farm Hospital, the hospital ships, and for the last year has been night superintendent at the Northern Hospital, Winchmore Hill, N., which post she leaves to take up her new appointment.

THE GROSVENOR HOSPITAL FOR WOMEN AND CHILDREN, VINCENT SQUARE, WESTMINSTER.—Miss Grace Elliott Coxon has been appointed Dispenser at this hospital. Miss Coxon was trained at the College of Pharmacy for Ladies in Westbourne Park Road, and holds the assistants' certificate of the Society of Apothecaries.

NORTHERN HOSPITAL, WINCHMORE HILL, N.—Miss E. Firth has been appointed Night Superintendent at this hospital, where she has held the post of charge nurse for three years. She was trained at the Mill Road Infirmary, Liverpool.

### An Unexpected Cure.

NURSE EDA WORTABEL sends us the following record of a case which she has nursed with care and evidently with much interest. We think, however, that the doctor, perhaps, tried to comfort the nurse in her distress, and that too much importance must not be attributed to all he said about the carbolic acid episode. It is an interesting record, especially as showing what vitality some children possess, and how firmly we must cling to hope in treating them:—

"Perhaps the following case may interest other nurses. It was a boy of five, his parents were strong and healthy, but his elder sister had lateral spinal curvature. At the age of two and a half years he showed signs of cordosis, and was put in plaster of Paris jacket, but he only grew worse, and finally sickened and developed an enormous abscess in the left groin. He was admitted into the hospital, but grew weaker and feebler daily, until the case was looked upon as hopelessly incurable and the child's life only a matter of a few weeks. The parents, hearing this, took him home, and applied for my services. He was a beautiful child, with a sweet, refined face and disposition which won him the name of 'Angel,' and it seemed almost cruel to disturb the wasted little form which lay so helpless and listless in its cot. The first few days I got nothing but a smile from him, but one morning he said to me, 'Nurse, I love you, because I think you are going to make me well.' 'Would you like to get well?' I said. 'I should; I don't want to be an angel, I want to be a man and walk.' This made me think. If I had an influence over the child, and he had faith in me, why not try massage. Gaining the doctor's permission, I started at once, and to my joy he seemed quite to delight in it—especially the back movements. Day by day he grew brighter and stronger, and he used to go into peals of laughter. I used to rub his back with methylated spirits to prevent bed sores. One morning I made a terrible mistake, and used carbolic instead. In one instant the whole back turned crimson. The child screamed, and I realised what I had done. I smeared the back with boracic vaseline, dressed it, and went to the doctor, who, seeing my distress, said, 'Never mind, nurse, doctors have done worse things; don't take it to heart, that child could never live. You have done all that can be done; I will see him later.' I returned to my little patient, who was still crying. He threw himself into my arms and soon fell off to sleep with his head on my shoulder. A large blister formed on the back, and a considerable quantity of fluid escaped. I dressed the place with boric ointment at first, and then zinc ointment. It healed, the child progressed. I continued massage again, and within three months of my undertaking the case the little fellow walked. The doctor said the carbolic had acted as a beneficial blister, and that the recovery was nothing less than miraculous."

### Appointments.

DEVON AND EXETER HOSPITAL, EXETER.—Miss E. Bath, who has for some time been a ward sister in the above hospital, has been appointed Assistant Matron from March 1st next. She was trained at St. Thomas's Hospital, London, and was appointed a ward sister of the Devon and Exeter Hospital in August, 1893.

BRISTOL NURSES' INSTITUTION AND NURSING HOME.—Miss Annie Thompson-Hill was appointed Lady Superintendent here on February 13th. She was trained at Pendlebury Children's Hospital for two years, and at King's College Hospital, London, for three years. For the last six years she has been assistant at the London Hospital.

CARMARTHENSHIRE INFIRMARY.—On the 14th inst. Miss Linda Ditcham was appointed Matron here. She was trained at Guy's Hospital, London. She then became charge nurse, and subsequently matron of Cumberland Infirmary, Carlisle.

### For Reading to the Sick.

### THE SEASON OF LENT.

### Verses.

THE man who without murmuring endures
Even the little sufferings of sustained
Exertion or privation (hourly cures,
For the disease of self, if self-ordained),
Hath in his aspect something which allures,
That sentiment our nature hath retained
Of the sublime—a sentiment that speaks
As do the cataracts to the mountain peaks.

—*Lytton.*

O perfect Love that 'dureth long!
Dear growth that shaded by the palm,
And breathed on by the angels' song,
Bloom on in Heaven's eternal calm.
How great the task to guard thee here
Where wind is rough and frost is keen,
And all the ground with doubt and fear
Is chequered birth and death between.

—*J. Ingelow.*

O Thou Who keep'st the Key of Love,
Open Thy fount, Eternal Dove,
And overflow this heart of mine,
Enlarging as it fills with Thee
Till in one blaze of charity,
Care and remorse are lost, like motes in light divine!
Till as each moment wafts us higher—
By every gush of pure desire,
And high breath'd hopes of joys above,
By every secret sigh we heave,
Whole years of folly we outlive,
In His unerring sight Who measures Life by Love.

—*Keble.*

I make me cords to hold from wrong,
And bind my will by purpose strong,
But my resolves as cords of tow
Before the strength of passion go,
Like hempen bonds, which flames o'er-run,
Or icy streams before the sun. . . .
Lord, who hast ta'en us by Thy hand,
'Tis only by Thy strength we stand.

—*J. Williams.*

### Reading.

No man is perfect and holy, but he has sometimes temptations; and altogether without them we cannot be.—*Thos. à Kempis.*

Let not a man trust his victory over his nature too far; for nature will lie buried a great time, and yet revive upon the temptation.

### A Prayer for the Season.

O, most mighty God, and merciful Father, Who hast compassion upon all men, and hatest nothing that Thou hast made; receive and comfort us who are grieved and wearied with the burden of our sin; give us unfeigned repentance for all the errors of our life past, and steadfast faith in Thy Son Jesus; that our sins may be done away by Thy mercy, and our pardon sealed in heaven, before we go hence and are seen no more.

As there are seasons in the year so are there times and seasons in man's life, and now is the solemn season of Lent when the Church bids every Christian man and woman draw aside for a while from the calls of the world and turn their thoughts on the things that perish not. But with the abstinence, the self-searching, and the self-examination that the Church requires, is there not always the glad thought that this season of expiation is but to fit us to share in the rejoicings of the glad Eastertide?—*R. M. Knowles.*

### Wants and Workers.

MISS MARCON, Norfolk House, Beaconsfield, Bucks, will be glad to hear of any nurse willing to dispose of the copies of the "Nursing Mirror" containing the cookery and special diet articles which have appeared during the past two years.

### Travel Notes.

By Our Travelling Correspondent.

### XI.—PAU AND THE PYRENEES.

We must shift our ground for a time and change the scene to the Pyrenees, a country the beauty of which is in no way behind that of the Riviera, though of a totally different character.

At different times I have spent nearly two years in the beautiful town of Henry IV., who was born in its splendid château, nursed in the neighbouring hamlet of Billère, and who to his death loved to be called the "Bearnais." The climate is delicious, and free entirely from what makes the Riviera objectionable at times. The mistral is unknown; occasionally a mysterious species of sirocco blows, which the natives speak of as the "Spanish wind," but it is harmless, though extremely disagreeable; it is very sudden in its advent, and blows with great force and extraordinary heat, but only lasts a few hours, not often more than about one.

### Climate.

There is a remarkable stillness in the atmosphere of Pau, which is found to be very advantageous in many varieties of chest troubles, and rheumatic patients derive great benefit from a residence there, also those suffering from bronchitis without further lung complication. It is peculiarly adapted for cases of incipient consumption. A friend of mine aged 16, and suffering from the earliest advances of consumption, went there in October in a most precarious and threatening state of health, and remained a fixture till the following September, braving the extreme heat of the summer months, and apparently deriving great benefit from it. In September he moved to Bermuda to join his parents, and is a strong and healthy man now. We troubled him with no doctor but tried the fresh air cure—driving, walking, and sitting out all day.

### The Journey is an easy one, viâ Dover, Calais, Paris, and Bordeaux, first-class, £6 11s.; and second, £4 9s. 11d. If you sleep in Paris go on by the 11.18 the next morning, which will land you in Bordeaux at ten o'clock in time for a good night's rest if you desire it, going on the next morning at almost any time you like. The length of time between Bordeaux and Pau is about five hours. There is a much cheaper way to reach Pau, and for some invalids (if good sailors) very advantageous, namely, by sea to Bordeaux, first-class, £3 11s. They are good boats and the time occupied is three days.

### Expenses of Residence.

These are not so heavy as on the Riviera, still accommodation in good hotels is sufficiently dear. The Hotel Gassion is an ideal house with a splendid view across the mountains, the same which I give you in my illustration. If a visitor is too ill to be continually in the open air it is of course essential that he should have rooms looking to the south, which immediately sends up the price, but if he is sufficiently robust to spend most of his day out of doors, a bedroom with a different aspect may be permitted, and if you are making a considerable stay the proprietor will generally met your terms. There are many splendid hotels and plenty of apartments, but I really think little is gained by taking apartments, and sometimes they are the cause of considerable trouble. The fashionable world seldom stays in Pau beyond April or May at the latest; the heat and the exigencies of society take them away, but they miss the loveliest season of all if they fail to see the Pyrenees in June.

### Social Attractions.

It is a very gay place, and in the season swarms with visitors, principally English and American. There are splendid golf links above Billère; there is tennis and polo to be had, as well as a large court for the local game of Paume, and there is a very good subscription pack of hounds. Also the theatre is good, and first-rate companies play there, and most excellent concerts are given twice a week in the Casino. The Garrison Band plays twice a week in the afternoons on the Place Royale, and nearly every night also. One occupies little tables at

Pic du Midi d'Ossau.

the various restaurants, and the whole thing is most enjoyable. The Place Royale itself is one of the most extended promenades in Europe, and during the last three years immense improvements have been made connecting it with the magnificent Royal Park on one side and the Park Beaumont on the other. Before the pedestrian lies the splendid panorama of the Pyrenees, at his feet the rushing Gave. By this feat of engineering invalids may enjoy a sheltered and uninterrupted walk of some two miles, always with the exquisite mountain range in front. In the winter the ever-changing effects are most entrancing, but as warm weather approaches the mountains are far less distinct, and become flat and less changeable from being always partially shrouded in heat mist. The Pic du Midi d'Ossau is always striking under all aspects, and there is a beauty in the Pyrenees that one misses in the Alps, namely, the lower range of hills at the base of the snow mountains, called Coteaux. These are clothed in the richest vegetation, and about their feet grow lovely fruit trees, peaches, pears, and apples, which in the warm spring weather burst out suddenly and make a veritable fairyland.

### The Royal Castle.

The castle itself, the gardens and moat that surround it, and the Royal Park adjacent, are best described by the word stately. They seem to have nothing in common with modern

**days, but to be peculiarly suited to those times when majesty clothed itself in ermine, and did not mix with the common herd. The restorations have on the whole been undertaken with good taste. Louis Philippe was greatly interested in this splendid relic of royalty, and did much to furnish it suitably. The grand staircase with its splendid doors is most impressive, and there are many objects, such as the cradle formed of a huge tortoise-shell, which rocked the slumbers of the great Henry, that one gazes upon with great interest. The tower of Gaston de Foix is imposing; unlike the rest of the building, it is entirely composed of tiles, which produce a rich purple-red effect, very striking when one sees it by sunset; in this tower are the principal oubliettes and dungeons. Next week I shall tell you of some buildings of historical interest in the town, and some of the numerous excursions.**

### TRAVEL NOTES AND QUERIES.

Rules in regard to Correspondence for this Section.—All questioners must use a pseudonym for publication, but the communication must also bear the writer's own name and address as well, which will be regarded as confidential. All such communications to be addressed "Travel Editor, 'Nursing Mirror,' 28, Southampton Street, Strand." No charge will be made for inserting and answering questions in the inquiry column, and all will be answered in rotation as space permits. If an answer by letter is required, a stamped and addressed envelope must be enclosed, together with 2s. 6d., which fee will be devoted to the objects of the "Hospital Convalescent Fund." Any inquiries reaching the office after Monday cannot be answered in "The Mirror" of the current week.

Pisa (Arbutus).—1. It is considered a good place for those suffering slightly from pulmonary complaints. It would not be my advice to go there, the Tramontana blows sometimes with cruel force, and I have recollections of bitter days. Spezzia is a charming place, and very sheltered. 2. More capabilities for the artist.

St. Malo (Elfrida).—Not a place for an invalid, as it is a walled town, shut into a very confined space, and sanitary arrangements are deficient. Why not try Dinard?

Pau (Boabdil).—Delightful from September to May, after that it is too hot for most people, and there is a general exodus to the mountains. Apartments are rather dear, and so are the hotels, but, like all other places, more moderate accommodation is to be found with careful research.

Lerici (Tel-el-Kebir).—A most lovely place, but I should stay at Spezzia by preference, which is very near.

Majorca (Beast of Burden).—I am told that the cruelty you speak of is grossly exaggerated, but I cannot answer from personal experience.

Brussels (E. P. P.)—You have forgotten our rule to use a pseudonym. It is certainly cold, but it greatly depends on the state of the delicate visitor. Consult your doctor and let me hear again.

*For Travel Advertisements see page xxi.*

### Notes and Queries.

**The contents of the Editor's Letter-box have now reached such unwieldy proportions that it has become necessary to establish a hard and fast rule regarding Answers to Correspondents. In future, all questions requiring replies will continue to be answered in this column without any fee. If an answer is required by letter, a fee of half-a-crown must be enclosed with the note containing the enquiry. We are always pleased to help our numerous correspondents to the fullest extent, and we can trust them to sympathise in the overwhelming amount of writing which makes the new rules a necessity.**

**Every communication must be accompanied by the writer's name and address, otherwise it will receive no attention.**

*Presentations.*

(206) We would like to know the reason why the account of the presentation sent in the week before last has not appeared?—*The Nurses.*

It has been deemed advisable not to insert the account of any presentations excepting those taking place on the occasion of bidding farewell to the recipient.

*Hypodermic Syringes.*

(207) "A. G. W." would be glad to know if there is a hypodermic syringe made that any maker's hypodermic needle can be used upon, where it can be bought, and the price.

There is no one syringe for every needle, because nothing is so satisfactory as a well-made screw, and each maker makes a particular thread, so that the syringe made by him can only be used with the needle intended for it. A syringe which would even adapt itself to a comparatively few makes of needles would necessarily have to have a plug, and this style is generally adap'ed for syringes of cheap make, and has manifold disadvantages. Unless both the nozzle of a syringe and the cap of the needle are very carefully turned there is always a large amount of leakage, though perhaps this is not visible when tried by merely passing a little water or solution through, yet directly there is any resistance, which is generally formed by the needle being inserted in the skin, half of the solution intended to be given hypodermically escapes in the joints. You might find the syringe described in page 100 of The Hospital for November 6th, 1897, introduced by Messrs. Parke, Davis, and Co., 21, North Audley Street, Grosvenor Square, W., or Messrs. W. H. Bailey and Son's (38, Oxford Street) Aseptic Seamless Hypodermic Needles useful.

*Sisters at Home and Abroad.*

(208) Could you kindly inform me the address of the publishing office of "The Sisters at Home and Abroad"?—*W. W. F.*

We have made inquiries, and no trace of this publication can be found in any of the publishers' catalogues.

*Operating Table.*

(209) Can you tell me where, and at what price, I could get an operating table for a private hospital? I have heard of one with a hot-water appliance for keeping patient warm during operation. Could you tell me where it is sold and the price?—*A. J.*

Messrs. Maw, Son, and Thompson (London) sell such a table, price from £15 15s. Messrs. Down and Son (London) have also one of the kind. Write to both makers for an illustrated price list.

*Patents and Bottles.*

(210) Will you kindly tell me the advantage and cost of patenting a medicine, also the cost of a licence to sell the same containing alcohol? Where could I best obtain bottles with boxes suitable for sending through the post?—*Patentee.*

You will find particulars relating to patent medicines on page 432 Whitaker's Almanack, and the British and Foreign Bottle Company, 107, Cannon Street, E.C., might quote prices for bottles.

*Travelling Nurse.*

(211) Would you kindly tell me what is the best time of year to advertise for a patient going out to South Africa? I am hoping to go out, and thought if I could get an invalid or some children in return for the passage money it would be most helpful. I am fully trained.—*South Africa*

Patients with chest complaints of course try and avoid the winter in England, but others travel at all seasons. The United British Women's Emigration Society, Imperial Institute, S.W., helps nurses, amongst others, to emigrate.

*Probationer.*

(212) I have a great desire to be a professional nurse. I am not 20 until September of this year, so that I am unable to enter any of the recognised training schools which you are mentioning in your columns under the heading of "Training in the Provinces," for at least another year and a-half. But if I succeeded in being admitted as "probationer" in any of the public hospitals, and underwent say three or four years training, should I be on the same footing as one that had been training at a recognised school? Would it be in any way detrimental to me? Also, my friends tell me that the profession is overstocked now. I should very much like your opinion on the matter. I am of a good height, and perfect health.—*Edith M. A.*

(213) I am anxious to become a hospital nurse. Will you kindly inform me at what age I am eligible to become a probationer, as I am now 17 years of age?—*F. M. F.*

"Edith M. A." may be quite sure that no public hospital that is a recognised training school will receive her as probationer yet. If both correspondents consult "The Nursing Profession: Where and How to Train" (Scientific Press, London, price 2s.), they will find that one or two special or cottage hospitals take probationers as young as 18 years of age, and that every added year to their age opens more doors for training. They must, however, be prepared to find that all their work before they enter a recognised training school for general nursing goes for nothing in their certificates, and that previous training is an obstacle to their admission into many schools. Some branches of nursing are over-crowded, but there is always room for sensible, strong, well trained women, especially in Poor-law work.

*District Nursing.*

(214) Can you oblige me by informing me where and how I can obtain a knowledge in district nursing? I am unable to pay for training, but have a great desire to become a nurse.—*E. Sims.*

Advertisements are constantly appearing in the "Mirror" for probationers, who are to receive training in return for service. If possible, enter for a three years' course in a recognised training school; for names of schools and conditions of training see "The Nursing Profession: Where and How to Train," price 2s., from the Scientific Press, London, W.

*Homes for the Aged.*

(215) Could you tell me of some home or institute where a friendless and feeble old lady who is nearly blind could be received and cared for for a small fee?—*E. M. B.*

(216) Will you kindly tell me whether there is any institution or home that would receive a domestic servant past work (age 69), or must I seek a private home for her? Ten shillings could be paid per week with her.—*Nurse Edith.*

There are a number of homes where persons who can pay a small sum weekly can be received, but care must be exercised in selecting a suitable one. If "E. M. B" and "Nurse Edith" consult Burdett's "Hospitals and Charities" they will find lists of homes supported either wholly or in part by charity. For other homes the list approved by the Y.W.C.A. and compiled by Miss Coles, 188, Ebury Street, price 1½d., is very useful.

*District Training.*

(217) Could you let me know if the enclosed advertisement is a bona-fide training home, and if the training would be sufficient for a district nurse?—*M. W.*

We cannot undertake to criticise institutions. The home is apparently a private one as its name does not appear in the "Nursing Profession." "M. W." should therefore find out how long it has been established and what is the method of training. As the training is only for three months the certificate could hardly be of any value.

*Vegetarian.*

(218) Will you please give me the address of any hospitals worked on vegetarian principles; I understand that there are three at least. I wish to train as a nurse in one.—*E. O*

Oriolet Cottage Hospital and Convalescent Home, Loughton, Essex, is conducted on vegetarian principles. Probationers are trained for two years.

## Figures and Tables

**Figure f1:**